# Myrtenol Attenuates MRSA Biofilm and Virulence by Suppressing *sarA* Expression Dynamism

**DOI:** 10.3389/fmicb.2019.02027

**Published:** 2019-09-04

**Authors:** Anthonymuthu Selvaraj, Thangaraj Jayasree, Alaguvel Valliammai, Shunmugiah Karutha Pandian

**Affiliations:** Department of Biotechnology, Alagappa University, Karaikudi, India

**Keywords:** Alamar blue, biofilm, eDNA, myrtenol, Methicillin-resistant *Staphylococcus aureus*, PBMC, *sarA*, transcriptional analysis

## Abstract

Methicillin-resistant *Staphylococcus aureus* (MRSA) is a deleterious human pathogen responsible for severe morbidity and mortality worldwide. The pathogen has attained high priority in the World Health Organization (WHO) – Multidrug-resistant (MDR) pathogens list. Emerging MDR strains of *S. aureus* are clinically challenging due to failure in conventional antibiotic therapy. Biofilm formation is one of the underlying mechanisms behind the antibiotic resistance. Hence, attenuating biofilm formation has become an alternative strategy to control persistent infections. The current study is probably the first that focuses on the antibiofilm and antivirulence potential of myrtenol against MRSA and its clinical isolates. Myrtenol exhibited a concentration-dependent biofilm inhibition without causing any harmful effect on cell growth and viability. Further, microscopic analysis validated the biofilm inhibitory efficacy of myrtenol against MRSA. In addition, myrtenol inhibited the synthesis of major virulence factors including slime, lipase, α-hemolysin, staphyloxanthin and autolysin. Inhibition of staphyloxanthin in turn sensitized the MRSA cells to healthy human blood and hydrogen peroxide (H_2_O_2_). Notably, myrtenol treated cells were deficient in extracellular DNA (eDNA) mediated autoaggregation as eDNA releasing autolysis was impaired by myrtenol. Biofilm disruptive activity on preformed biofilms was observed at concentrations higher than minimum biofilm inhibitory concentration (MBIC) of myrtenol. Also, the non-cytotoxic effect of myrtenol on human peripheral blood mononuclear cell (PBMC) was evidenced by trypan blue and Alamar blue assays. Transcriptional analysis unveiled the down-regulation of global regulator *sarA* and *sarA* mediated virulence genes upon myrtenol treatment, which is well correlated with results of phenotypic assays. Thus, the results of the present study revealed the *sarA* mediated antibiofilm and antivirulence potential of myrtenol against MRSA.

## Introduction

Globally, bacterial infections are treated and also prevented by antibiotics. The extensive and inappropriate usage of antibiotics induces selective pressure on the bacterial community in developing such antibiotic resistance. Hence, emergence of MDR decreases the potential of antibiotics against bacteria (Qiao et al., 2018). According to WHO, antimicrobial resistance is a major concern to global public health (WHO, 2015). Methicillin-resistant *Staphylococcus aureus* (MRSA) is one among the highly concerned MDR pathogens and it has been categorized as a high priority MDR pathogen by WHO (2017).

In earlier days, most of the MRSA infections were associated with hospitalized patients and hence, MRSA strains isolated from hospital settings are named as healthcare-associated MRSA (HA-MRSA). Over the course of time, the prevalence of MRSA has changed. MRSA strains have been identified even in patients not previously hospitalized and are named as community-associated MRSA (CA-MRSA) (Boswihi and Udo, 2018). Generally, MRSA is known to cause skin and soft tissue infections, bacteremia, infective endocarditis, osteomyelitis, and pneumonia. Additionally, MRSA is predominantly involved in indwelling catheter infections, prosthetic devices and implant associated infections (Hassoun et al., 2017; Lee et al., 2018).

One of the major and pivotal characteristics of MRSA is the ability to form biofilm on biotic and abiotic surfaces, which is the prime cause for its resistance and persistence (Savage et al., 2013). MRSA biofilm is made of self-produced extracellular polymeric substances which comprise of polysaccharides, proteins and eDNA. Biofilm provides protection to MRSA from host innate immune response and acts as barrier to the entry of antibiotics (Lister and Horswill, 2014). MRSA is armored with many cell surfaces and secreted virulence factors. Cell surface virulence factors are majorly involved in adherence and persistence and include capsular polysaccharides, staphyloxanthin and microbial surface components recognizing adhesive matrix molecules (MSCRAMMs) such as staphylococcal protein A (SpA), fibronectin-binding proteins (FnBPs), collagen-binding protein (Cna) and clumping factors (ClfA and ClfB). Secreted virulence factors are often associated with tissue invasion and disease progression which include staphylococcal enterotoxins, α-hemolysin, leukocidin and virulence enzymes such as lipases, nucleases, proteases, aureolysin, hyaluronidase, and staphylokinase (Bien et al., 2011; Al-Mebairik et al., 2016). These virulence factor secretion and biofilm formation are significantly mediated by global regulatory systems such as accessory gene regulator (*agr*), staphylococcal accessory element (*sae*) and also by staphylococcal accessory regulator A (*sarA*) (Bronner et al., 2004). A DNA binding protein sarA encoded by *sar* locus has been reported to play a significant role in regulating the expression of virulence factors in MRSA. SarA modulates the virulence gene expression by binding to the intergenic space between P2 and P3 promoters of *agr* regulon. Though crosstalk between *agr* and *sarA* is well documented, *sarA* controls the expression of several important adhesion genes through *agr* independent mechanism. Hence, being a master regulator of biofilm and virulence genes, *sarA* currently stands as a therapeutic target for drug development. In addition, *sarA* inhibitors are well known to inhibit biofilm formation by *Staphylococcus aureus* (Arya et al., 2015).

Antibiofilm therapeutic approach is an alternative strategy to overcome antibiotic resistance. The target of novel therapy is to inhibit biofilm formation and virulence factors production instead of killing the bacteria and hence it excludes the selection pressure on bacteria, thereby avoiding resistance development (Chung and Toh, 2014). Recently, several studies culminated in the identification of antibiofilm agents from natural resources against MRSA such as quercetin and tannic acid from *Acidia japonica* (Lee et al., 2013), stilbenes (Lee et al., 2014), alizarin from madder plants (Lee et al., 2016), α-mangostin from *Garcinia mangostana*, (Phuong et al., 2017), and nerolidol from *Pogostemon heyneanus* (Rubini et al., 2018a). The objective of the current study is to determine the antibiofilm and antivirulence agent from natural resources against MRSA. In the present study, ten different phytochemicals were screened against MRSA for their antibiofilm potential, among which myrtenol exhibited strong antibiofilm activity. Myrtenol (PubChem CID:10582), a bicyclic alcohol mono-terpene plant derivative commonly known for its pleasant aroma, is often used as a fragrance ingredient in a wide range of cosmetic and non-cosmetic products (Bhatia et al., 2008). This bioactive metabolite is found in the essential oil of numerous medicinal plants such as *Myrtus communis* (Baharvand-Ahmadi et al., 2015), *Tanacetum vulgare* (Mockute and Judzentiene, 2003), *Turnera diffusa* (Alcaraz-Meléndez et al., 2004), *Anemopsis californica* (Medina-Holguín et al., 2008), *Lippia multiflora* (Menut et al., 1995), *Rhodiola rosea*, *Paeonia lactiflora*, *Cyperus rotundus*, etc., (Gomes et al., 2017). In traditional medicine, myrtenol has been explored for the treatment of anxiety, gastrointestinal pain, inflammations and infections (Gomes et al., 2017; Sisay and Gashaw, 2017). Furthermore, several reports have confirmed these antimicrobial (Paknejadi and Foroohi, 2012), antioxidant (Coté et al., 2017), hypotensive, antinociceptive (Silva et al., 2014) and acetylcholinesterase activities (Miyazawa and Yamafuji, 2005) of myrtenol. In spite of numerous biological activities, the antibiofilm potential of myrtenol is yet to be explored. Hence, the present study is aimed at investigating the efficacy of myrtenol on MRSA biofilm formation and virulence factors production and also at unveiling its mode of action through a transcriptional approach.

## Materials and Methods

### Ethics Statement

In the present study, healthy human blood was used for blood survival and cytotoxicity assay and sheep blood was used for hemolysin production assay. The human blood sample was taken by a trained person from the healthy individuals and a written informed consent was obtained. The usage of the human blood sample and experimental methodology was assessed and approved by the Institutional Ethical Committee, Alagappa University, Karaikudi (IEC Ref No: IEC/AU/2018/4). The sheep blood used in the study was collected from the Karaikudi municipality slaughterhouse. There is no specific ethical permission since sheep blood is discarded in the slaughterhouse.

### Bacterial Strains and Culture Conditions

The MRSA strains used in this study are listed in Table 1. The clinical strains were isolated and identified by Gowrishankar et al. (2012). All the strains were cultured on tryptic soy agar (TSA, Hi-Media, India) at 37°C for 24 h and stored at 4°C for future use. For *in vitro* assays, MRSA was cultured in tryptic soy broth with 0.5% sucrose (TSBS) and incubated in an orbital shaker (160 rpm) at 37°C overnight. One percent of overnight MRSA culture was used for all assays.

**TABLE 1:** List of strains used in this study.

**Strain name**	**Details**
MRSA	ATCC 33591
MRSA 395	GenBank ID: JN390832
MRSA 410	GenBank ID: JN315150
MRSA 44	GenBank ID: JN31514

### Phytochemicals

Stock solutions of phytochemicals were prepared as 10 mg ml^–1^ concentration and stored at 4^*o*^C for further use. Myrtenol, α-bisabolol, carvacrol, phytic acid and ethyl linoleate were procured from Sigma-Aldrich, India. Cineole, theophyline, borneol, oleic acid and α-pinene were procured from Alfa Aeser, India. Methanol (Sigma-Aldrich, India) was used to dissolve myrtenol, ethyl linoleate and theophyline. Ethanol (Sigma-Aldrich, India) was used to dissolve α-bisabolol, phytic acid, cineole, theophyline, borneol, oleic acid and α-pinene.

### Determination of Minimum Inhibitory Concentration (MIC) and Minimum Biofilm Inhibitory Concentration (MBIC)

Minimum inhibitory concentration of myrtenol was assessed by micro-broth dilution assay as described by the Clinical and Laboratory Standards Institute method (CLSI, 2018). Briefly, one percent of culture was added into a 24 well sterile micro-titer plate containing TSBS with different concentrations (25, 50, 100, 150, 200, 250, 300, 350, 400, 450, 500, 550, and 600 μg ml^–1^) of myrtenol. TSBS containing 20 μl of methanol and 10 μl of culture was considered as vehicle control and TSBS alone was used as negative control. After incubating at 37°C for 24 h, the plate was read at 600 nm (Spectramax M3, Molecular devices, United States) to determine the MIC of myrtenol. For the determination of MBIC, planktonic cells were discarded and the plate was washed with distilled water to remove unbound cells and air dried. Crystal violet (0.4%) solution was used to stain the biofilm cells for 10 min and washed again with distilled water to remove unbound stains and then allowed to air dry. The biofilm cells were destained using 20% glacial acetic acid and absorbance of crystal violet solution was read at 570 nm. The amount of biofilm was directly proportional to the Optical Density (OD) value of the crystal violet solution in the wells. The percentage of biofilm inhibition was calculated by the following formula: Biofilm inhibition (%) = [(Control OD_570__nm_ - Treated OD_570__nm_)/Control OD_570__nm_] × 100 (Singh et al., 2018).

### Microscopic Visualization of Biofilm Inhibition

For microscopic visualization, the biofilm was allowed to grow on glass slides (1 cm × 1 cm) in the absence and presence of myrtenol (75, 150, and 300 μg ml^–1^) as described in the MBIC assay. Followed by biofilm assay, glass slides were washed with distilled water to remove the unbound cells and biofilm was stained by crystal violet solution (0.4%). Distilled water was used to wash excess stain on glass slides and allowed to air dry. Light microscope (Nikon Eclipse Ti-S, Tokyo, Japan) was used to observe the biofilm on glass slides at the magnification of 400X. For confocal laser scanning microscopy (CLSM) analysis, acridine orange solution (0.1%) was used to stain the biofilm on glass slides and visualized under CLSM (Zeiss LSM-710, Carl Zeiss, Oberkochen, Germany) at the magnification of 200X.

### Ring Biofilm Inhibition Assay

The effect of myrtenol on air-liquid interface biofilm formation was qualitatively determined by ring biofilm assay for which glass tubes containing one percentage of MRSA in 2 ml of TSBS in the absence and presence of myrtenol with increasing concentrations (75, 150, and 300 μg ml^–1^) were incubated for 24 h at 37°C. Then, the planktonic cells in test tubes were discarded and washed thrice with sterile distilled water and air dried. Further, the test tubes were stained by crystal violet (0.4%) and washed thrice with distilled water to remove excess stain. Finally, test tubes were allowed to air dry in inverted position and then visually observed and photographed (Mathur et al., 2006).

### Alamar Blue Assay

The Alamar blue [Resazurin (7-Hydroxy-3H-phenoxazin-3-one 10-oxide)] assay was performed to assess the metabolically active cells in control and treated samples (Gargotti et al., 2018). Stock solution of Alamar blue (Sigma-Aldrich, India) at 6.5 mg ml^–1^ in 1X Phosphate buffered saline (PBS) was prepared separately. The control and myrtenol (75, 150, and 300 μg ml^–1^) treated cells were harvested and washed twice with PBS and resuspended in PBS. Cell suspension (0.9 ml) and Alamar blue substrate (0.1 ml) were mixed and incubated in the dark at 37°C for 4 h. Sterile PBS with Alamar blue substrate was used as blank. After incubation, the samples were centrifuged at 8000 rpm for 10 min and the fluorescent intensity of the supernatant containing the reduced Alamar blue was observed at 590 nm emission and 560 nm excitation wavelengths.

### Colony Forming Unit (CFU) Assay

CFU assay was performed to determine the cell count variation between control and myrtenol treated cells (75, 150, and 300 μg ml^–1^) of MRSA. Briefly, MRSA was grown in the absence and presence of myrtenol for 24 h at 37°C. After incubation, control and treated MRSA cells were serially diluted up to 10^–8^ and spread on a TSB agar plate.

### Growth Curve Analysis

Growth curve assay was performed to assess the influence of myrtenol (300 μg ml^–1^) on the growth of MRSA. MRSA was grown in the absence and presence of myrtenol at 37°C. At a regular interval of 1 h, OD was observed at 600 nm up to 24 h.

### Slime Production Assay

In order to assess the slime production, congo red agar (CRA) plates were prepared with TSB (3%), sucrose (3.6%), and agar (1.8%). The congo red dye (0.08%) was prepared separately and added to the agar medium. Control and myrtenol (75, 150, and 300 μg ml^–1^) treated MRSA culture were streaked on CRA plates and incubated for 24 h at 37°C (Gowrishankar et al., 2016).

### Lipase Assay

The para-nitro phenyl palmitate (pNPP) substrate was used to assess the lipid hydrolytic activity of cell free culture supernatant (CFCS) of control and myrtenol treated MRSA. Briefly, the lipase substrate solution was prepared by adding one volume of pNPP [0.3% pNPP in 2-propanol] to nine volumes of 50 mM Tris–HCl buffer (pH 8.0) containing sodium deoxycholate (0.2%) and gummi arabicum (0.1%). CFCS was obtained by centrifugation of MRSA cells cultured in the absence and presence of myrtenol (75, 150, and 300 μg ml^–1^). To 100 μl of CFCS, 900 μl of lipase substrate was added and incubated at 37°C for 1 h and centrifuged at 10000 rpm for 10 min. Then, the supernatant was collected and absorbance was read at 410 nm to calculate lipase inhibition using following formula: Lipase inhibition (%) = [(Control OD_410__nm_ - Treated OD_410__nm_)/Control OD_410__nm_] × 100 (Patel et al., 2018).

### Hemolysis Quantification Assay

Cell free culture supernatant was collected from control and myrtenol treated (75, 150, and 300 μg ml^–1^) MRSA strains as described earlier. Hemolytic activity was determined by incubating the CFCS with equal volume of sheep red blood cells (2% v/v in PBS) for 1 h at 37°C. After incubation, tubes were centrifuged at 3000 rpm for 10 min and the OD of supernatant was measured at 405 nm to determine the hemolytic activity using the following formula: Hemolysin inhibition (%) = [(Control OD_405__nm_ - Treated OD_405__nm_)/Control OD_405__nm_] × 100 (Kannappan et al., 2017).

### Autolysis Assay

To determine the effect of myrtenol on the autolysis property of MRSA, control and myrtenol (75, 150, and 300 μg ml^–1^) treated MRSA cells were harvested by centrifugation at 8000 rpm for 10 min and washed twice with ice cold PBS. Then, the cell pellet was resuspended in PBS containing 0.02% (v/v) Triton X-100. The cell suspensions were incubated at 37°C and lysis of cells was monitored by measuring the OD at 600 nm every 30 min for 3 h (Rowe et al., 2010).

### Extraction of eDNA

The amount of eDNA in MRSA biofilm was qualitatively analyzed by agarose gel electrophoresis. Briefly, MRSA was allowed to form biofilm in the absence and presence of myrtenol (75, 150, and 300 μg ml^–1^) on a 6 well polystyrene plate for 24 h at 37°C. Then, the planktonic cells were removed and biofilm was washed three times with PBS. The biofilm cells were scraped, resuspended in TE buffer (10 mM Tris, 1 mM EDTA) and vigorously vortexed for 1 h then centrifuged at 8000 rpm for 10 min. The supernatant was collected and the amount of eDNA present in the supernatant was separated by 1.5% (w/v) agarose gel electrophoresis (Kaplan et al., 2012).

### Autoaggregation Assay

MRSA culture was grown in 10 ml of TSB medium with and without myrtenol (75, 150, and 300 μg ml^–1^) for 24 h at 37°C. The cell pellet was harvested by centrifugation at 8000 rpm for 10 min, washed thrice with PBS and resuspended in 10 ml of PBS. Bacterial suspensions were transferred to glass tubes and allowed to stand without any disruption for 24 h and 200 μl of the upper portion was collected at 0, 3, 6, 12, and 24 h time points for measuring the absorbance at 600 nm. For visual observation, tubes were photographed (Tareb et al., 2013).

### Extraction of Staphyloxanthin

To assess the staphyloxanthin production, one percentage of MRSA was used to inoculate in 10 ml of TSBS with and without the myrtenol (75, 150, and 300 μg ml^–1^). After 24 h incubation, MRSA culture was centrifuged at 10000 rpm for 10 min. Then, the pellet was washed twice with PBS and resuspended in 2 ml of methanol. For methanol extraction, the tubes were kept in a shaking incubator for 24 h. After the methanolic extract, the samples were centrifuged at 10000 rpm for 10 min and the OD of the supernatant was measured at 465 nm. The percentage of staphyloxanthin inhibition was calculated by following formula: staphyloxanthin inhibition (%) = [(Control OD_465__nm_ - Treated OD_465__nm_)/Control OD_465__nm_] × 100 (Lan et al., 2010).

### H_2_O_2_ Sensitivity Assay

Control and myrtenol (75, 150, and 300 μg ml^–1^) treated MRSA cells were pelleted by centrifugation at 8000 rpm for 10 min and resuspended in PBS containing 1 mM H_2_O_2_ and incubated at 37°C for 1 h. Then, the cells were serially diluted, spread on the TSB agar plate and incubated at 37°C for 24 h. After incubation, viable cells were counted to determine the sensitivity of cells to H_2_O_2_ (Tatsuno et al., 2014).

### Blood Sensitivity Assay

The control and myrtenol (75, 150, and 300 μg ml^–1^) treated cell suspensions were prepared in PBS. The cell suspensions were mixed gently with healthy human blood at a ratio of 1:4 and incubated at 37°C for 1 h. After incubation, the samples were serially diluted and spread over TSB agar plate. The plates were incubated for 24 h at 37°C and colonies were manually counted to identify the sensitivity of cells to healthy human blood (Subramenium et al., 2015).

### Real-Time PCR (qPCR) Analysis

Total RNA was extracted from MRSA grown for 24 h in the absence and presence of myrtenol (300 μg ml^–1^) by Trizol method of RNA isolation and converted to cDNA using High-capacity cDNA Reverse Transcription Kit (Applied Biosystems Inc., United States) according to the manufacturers’ protocol. qPCR analysis was performed on thermal cycler (7500 Sequence Detection System, Applied Biosystems Inc., Foster, CA, United States) for the genes such as *sarA* (transcriptional regulator), *icaA* and *icaD* (involved in poly-beta-1,6-N-acetyl glucosamine biosynthesis), *agrA* (response regulator), and *agrC* (transmembrane protein), *fnbA* and *fnbB* (fibronectin binding proteins), *clfA* (clumping factor), *cna* (collagen binding protein), *hla* (alpha-hemolysin), *hld* (delta-hemolysin), *geh* (lipase), *altA* (autolysin), and *crtM* (involved in staphyloxanthin biosynthesis) using PCR mix (SYBR Green kit, Applied Biosystems, United States) at a predefined ratio. Cycle threshold (Ct) values of all the tested genes were normalized using that of housekeeping gene *gyrB* (gyrase) and expression was quantified by 2^(–ΔΔCt)^ method (Livak and Schmittgen, 2001; Gowrishankar et al., 2015). Primer sequences of the genes taken for study and qPCR conditions are given in Supplementary Tables S1 and S2, respectively.

### Mature Biofilm Assay

To determine the effect of myrtenol on mature biofilm, MRSA was allowed to form biofilm on glass slides and polystyrene surface of 24 well plate for 24 h at 37°C as mentioned previously. After the formation of mature biofilm, the planktonic cells were discarded and washed with sterile PBS to remove non-adhered cells. Fresh TSBS media and various concentrations of myrtenol (75, 150, 300, and 600 μg ml^–1^) were added to the specific wells and incubated for 24 h at 37°C. After incubation, glass slides were washed thrice with sterile PBS and stained for observation under light microscope and CLSM as described previously. For biofilm quantification, the plate was washed with distilled water to remove unbound cells and biofilm cells were stained with crystal violet (0.4%) solution and washed again with distilled water and then allowed to air dry. The biofilm cells were destained using 20% glacial acetic acid and absorbance of crystal violet solution was read at 570 nm (Chen et al., 2018).

### Peripheral Blood Mononuclear Cell (PBMC) Isolation

The blood sample (5 ml) was collected from a healthy human volunteer in a tube containing EDTA. Equal volume of lymphocyte separation medium (Histopaque-HiMedia, India) was taken in a fresh tube upon which the freshly collected blood was layered carefully and subjected to centrifugation at 2200 rpm for 30 minutes at 20°C. After removing the upper layer, the buffy coat mononuclear layer was carefully transferred to a fresh tube and washed using RPMI-1640 (Roswell Park Memorial Institute) medium at 2200 rpm for 15 min at 20°C. The pellet was resuspended in the complete medium [RPMI medium (HiMedia, India), 10% fetal bovine serum (HiMedia, India) and 1% Anti-Anti (Antibiotic-Antimycotic) solution (Invitrogen, United States)] and the cell viability was assessed through trypan blue exclusion assay. PBMCs were adjusted to 1 × 10^6^ cells ml^–1^ in complete medium for further experiments (Syad and Kasi, 2014).

### Cytotoxicity Analysis

Cytotoxicity was assessed using PBMC by Alamar blue assay and trypan blue exclusion assay. PBMCs were incubated in the presence of different concentrations of myrtenol (75, 150, 300, and 600 μg ml^–1^) at 37°C with 5% CO_2_ incubator for 24 h. Appropriate control, positive control (1mM H_2_O_2_) and vehicle controls (0.05% DMSO) were also maintained. The cell viability was assessed after 24 h by trypan blue exclusion assay (Strober, 1997) using the formula: Percentage of cell cytotoxicity = (Number of viable cells/Total number of cells) X 100 (Rajavel et al., 2017).

Alamar blue was prepared in PBS solution at 1 mg ml^–1^ concentration. The control and treated PBMCs were collected by centrifugation and washed twice with PBS and resuspended in PBS. To 90 μl of cell suspension, 10 μl of Alamar blue solution was added and incubated in dark at 37°C for 4 h. After incubation, the samples were centrifuged at 8000 rpm for 10 min and the fluorescent intensity of the supernatant was observed at 590 nm emission and 560 nm excitation wavelengths (Menezes et al., 2016).

### Statistical Analysis

All the experiments were carried out in three biological replicates with at least two technical replicates and values are presented as Mean ± standard deviation (SD). To analyze the significant difference between the value of control and treated samples, one-way analysis of variance (ANOVA) and Duncan’s *post hoc* test was performed with the significant *p*-value of <0.05 by the SPSS statistical software package version 17.0 (Chicago, IL, United States).

## Results

### Effect of Phytochemicals on Growth and Biofilm Formation of MRSA

The antibiofilm activity of ten phytochemicals was tested against MRSA at 300 μg ml^–1^. Out of ten phytochemicals, myrtenol showed strong antibiofilm activity (72%) at 300 μg ml^–1^. Among the remaining phytochemicals, cineole, carvacrol, α-pinene and α-bisabolol exhibited antibacterial activity whereas the rest of the phytochemicals at 300 μg ml^–1^ concentrations showed insignificant changes in cell density as well as biofilm formation of MRSA (Supplementary Figure S1).

### Determination of MIC and MBIC of Myrtenol

**FIGURE 1:**
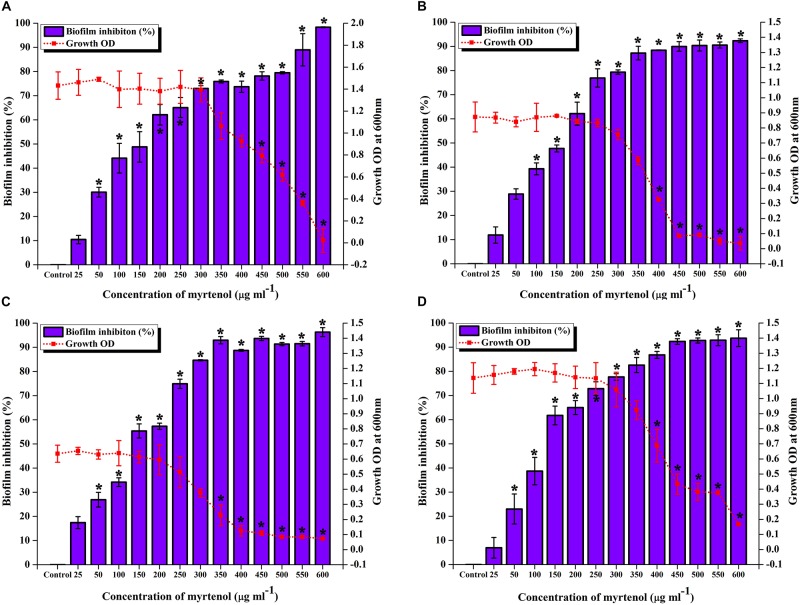
Effect of myrtenol at various concentrations (25–600 μg ml^–1^) on growth and biofilm of **(A)** MRSA ATCC 33591 **(B)** MRSA 395 **(C)** MRSA 410 **(D)** MRSA 44. Error bars indicate SD and asterisks indicate statistical significance (*p* ≤ 0.05).

To determine the MIC and MBIC of myrtenol against MRSA ATCC strain and clinical isolates, micro-broth dilution assay and biofilm assay was performed. The result exhibited myrtenol at 600 μg ml^–1^ which completely inhibited the growth of MRSA ATCC strain and clinical isolates. Therefore, MIC of myrtenol was fixed as 600 μg ml^–1^. Biofilm inhibition was assessed by crystal violet quantification assay. The results demonstrated the concentration dependent antibiofilm activity of myrtenol against MRSA strains. At 300 μg ml^–1^ concentration, myrtenol showed a maximum of 72% (MRSA ATCC-33591), 79% (MRSA-395), 85% (MRSA-410), and 82% (MRSA-44) of biofilm inhibition in MRSA strains. Inhibition of the growth was observed at concentrations higher than 300 μg ml^–1^ of myrtenol. An ideal antibiofilm agent should not affect the growth of the bacteria. Hence, 300 μg ml^–1^ was fixed as MBIC of myrtenol (Figures 1A–D).

### Microscopic Visualization of Biofilm Architecture

**FIGURE 2:**
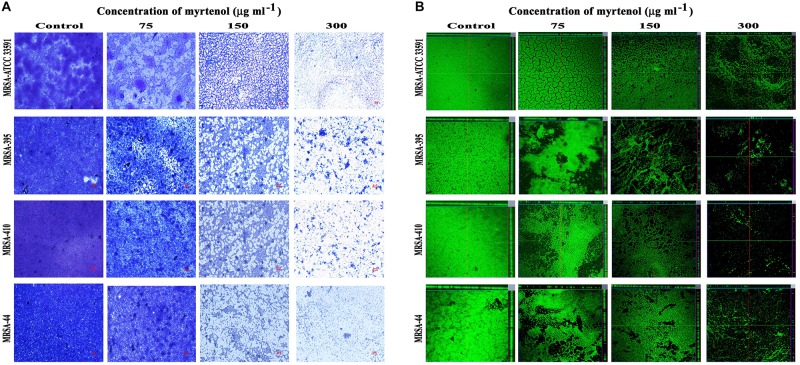
Light microscopic images **(A)** and CLSM images **(B)** demonstrating the antibiofilm potential of myrtenol at increasing concentrations against MRSA reference strain and clinical isolates.

Microscopic technique was used to qualitatively analyze biofilm architecture of MRSA strains. In light microscopic analysis, highly aggregated biofilm formation was observed in control samples. Whereas in treated samples, concentration dependent reduction in the biofilm covered surface area was observed (Figure 2A). The CLSM was used to assess the three-dimensional biofilm architecture of MRSA strains. Reduction in the thickness of biofilm formation was noticed in myrtenol treated samples when compared to that of control samples (Figure 2B).

### Antibiofilm Efficacy of Myrtenol Against MRSA

**FIGURE 3:**
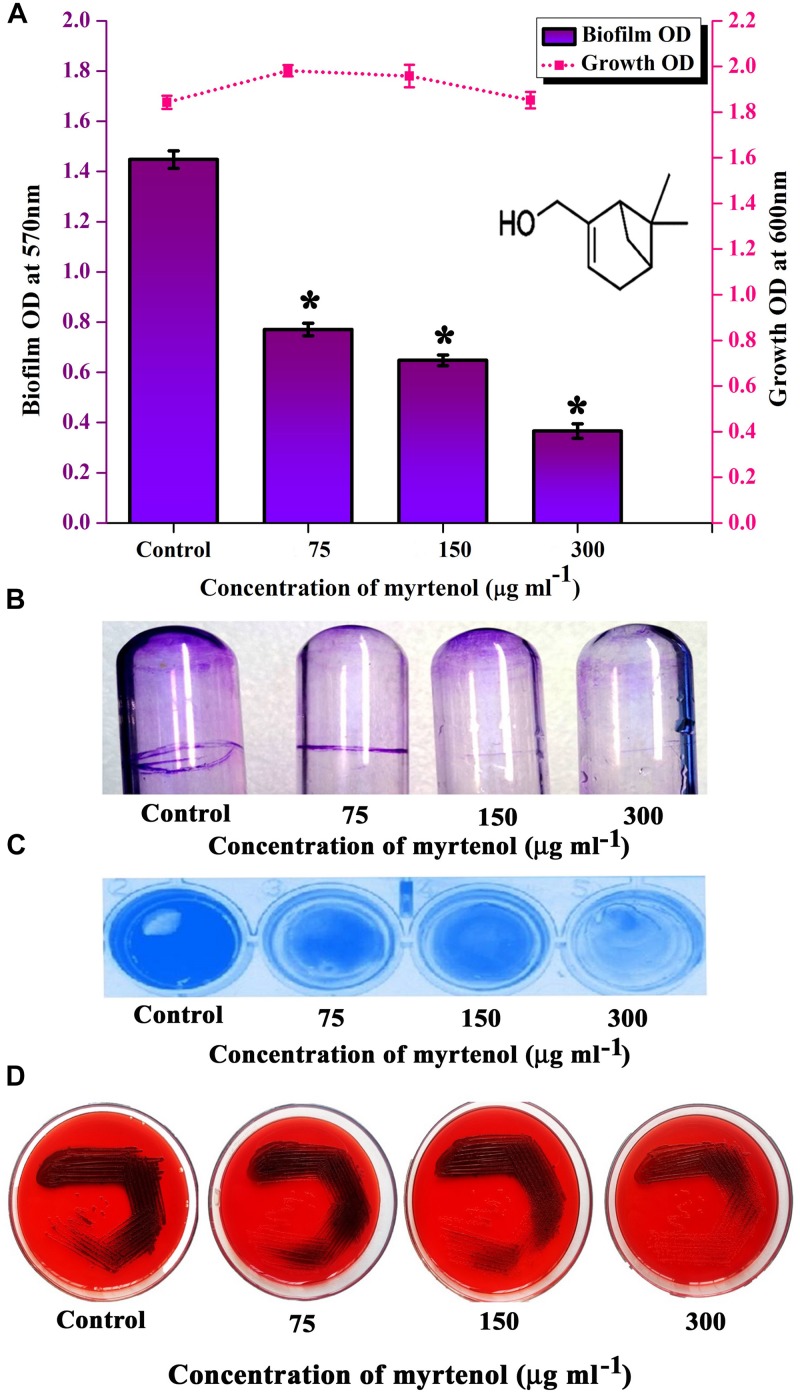
Analysis of antibiofilm potential of myrtenol at increasing concentrations against MRSA using **(A)** crystal violet quantification (The chemical structure of myrtenol was retrieved from PubChem CID: 10582). **(B)** Effect of myrtenol treatment on MRSA biofilm formation on glass test tubes and **(C)** polystyrene plate. **(D)** Qualitative analysis of slime synthesis of MRSA upon myrtenol treatment. Error bars indicate SD and asterisks indicate statistical significance (*p* ≤ 0.05).

MRSA ATCC strain was taken for further studies. Hence, the effect of myrtenol on biofilm reference strain was assessed. Myrtenol showed concentration dependent biofilm inhibitory activity against MRSA. At a concentration of 300 μg ml^–1^, myrtenol exhibited a maximum of 72% (*p* ≤ 0.05) biofilm inhibition in MRSA (Figure 3A). Growth OD values of control and treated samples did not reveal any significant difference up to 300 μg ml^–1^ concentrations of myrtenol (Figure 3A). In addition, myrtenol showed a concentration dependent reduction in ring biofilm formation on glass test tubes (Figure 3B) and surface biofilm formation on polystyrene plate (Figure 3C).

### Qualitative Analysis of Slime Production

In *S. aureus*, slime production is linked with biofilm formation. Hence the slime production was qualitatively analyzed by the CRA plate method. The results exhibited a reduction in the black coloration around the colonies of treated samples when compared to the colonies of control sample (Figure 3D).

### Effect of Myrtenol on Growth and Viability of MRSA

**FIGURE 4:**
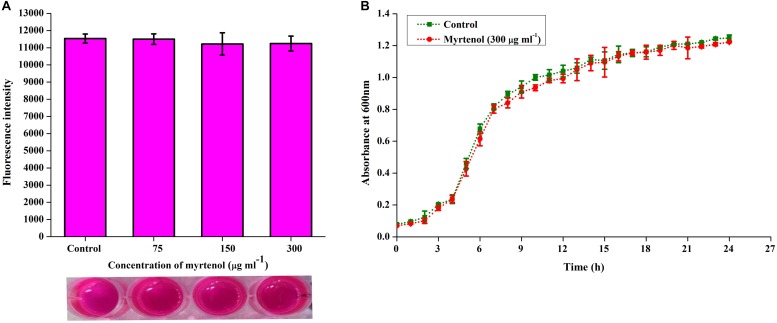
Effect of myrtenol on growth and viability of MRSA assessed by **(A)** Alamar blue reduction assay and **(B)** Growth curve analysis. Error bars indicate SD.

To determine the effect of myrtenol on growth and viability of MRSA, Alamar blue assay, CFU assay and growth curve analysis were performed. In Alamar blue assay, the fluorescent intensity of resazurin dye was found to be unchanged for control and myrtenol treated MRSA cells which directly confirms the non-antibacterial effect of myrtenol (Figure 4A). The CFU of MRSA control (4.8 × 10^8^ cells) and myrtenol treatment at MBIC (4.7 × 10^8^ cells) was found to be significantly unchanged (Supplementary Figure S2). Furthermore, the growth curve analysis of MRSA also showed no significant change in myrtenol treated sample compared to control culture (Figure 4B).

### Quantification of Lipase and Hemolysin Production

Extra-cellular lipase and hemolysin are important virulence factors produced by *S. aureus* to degrade the phospholipid bilayer of host cells and erythrocytes of host blood respectively. The results indicated significant reduction up to 64% of lipase and 62% of hemolysin production upon treatment with myrtenol at MBIC (*p* ≤ 0.05) (Supplementary Figure S3). In addition to ATCC strain, the effect of myrtenol on hemolysin production was assessed for clinical isolates and results showed a concentration dependent reduction in hemolysin production (Supplementary Figure S4).

### Autolysis Assay

**FIGURE 5:**
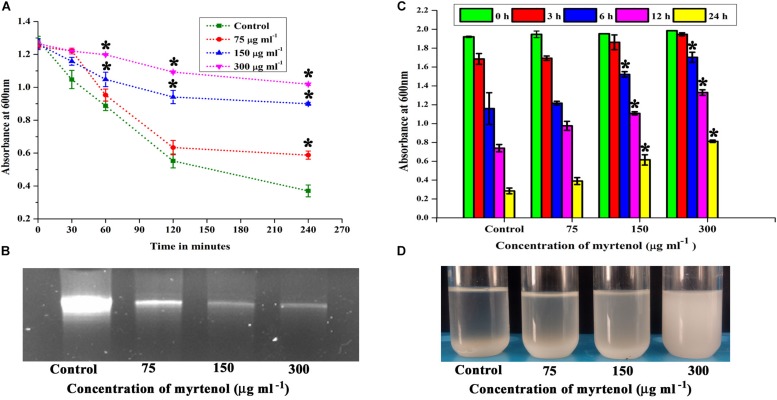
Effect of myrtenol on **(A)** autolysis, **(B)** eDNA synthesis and **(C)** autoaggregation of MRSA. **(D)** Photographs showing the pattern of autoaggregation upon myrtenol treatment. Error bars indicate the SD and asterisks indicate statistical significance (*p* ≤ 0.05).

Autolysin has been reported as an essential factor for *S. aureus* biofilm formation. Hence, the effect of myrtenol on autolysis of MRSA was spectrophotometrically measured. The results showed the inhibition in autolysis activity of myrtenol (75, 150, and 300 μg ml^–1^) treated cells, whereas in the control cells, highly active autolysis was observed (Figure 5A).

### Qualitative Analysis of eDNA Production

The quantity of eDNA released by MRSA was assessed in the absence and presence of myrtenol (75, 150, and 300 μg ml^–1^). The decrease in the amount of eDNA release was observed in myrtenol treated cells when compared to the amount of eDNA released by control cells (Figure 5B).

### Autoaggregation Property

Autoaggregation is an important process in *S. aureus* biofilm formation. Thus, the effect of myrtenol on the autoaggregation property of MRSA was evaluated. Interestingly, the autoaggregation of MRSA was reduced due to the addition of myrtenol in the growth medium. The significant reduction in autoaggregation was observed in myrtenol (75, 150, and 300 μg ml^–1^) treated samples compared to the control sample (Figure 5C). Autoaggregation was visually observed after incubating tubes for 24 h in static condition (Figure 5D).

### Staphyloxanthin Synthesis

**FIGURE 6:**
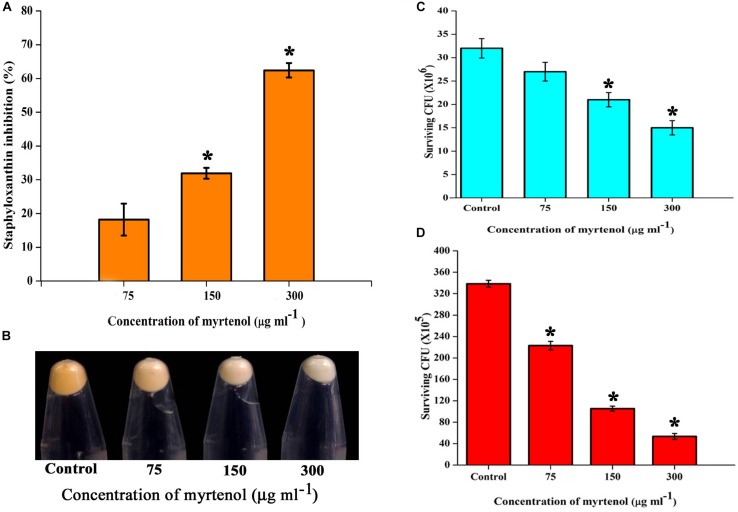
**(A)** Inhibitory effect of myrtenol on staphyloxanthin production of MRSA cells. **(B)** Photographs showing the inhibition of staphyloxanthin. Effect of myrtenol treatment on survival of MRSA in **(C)** H_2_O_2_ and **(D)** healthy human blood. Error bars indicate SD and asterisks indicate statistical significance (*p* ≤ 0.05).

Staphyloxanthin synthesis of myrtenol treated and untreated cells was evaluated quantitatively and qualitatively. Staphyloxanthin production was inhibited in a concentration dependent manner up to 20−65% (*p* ≤ 0.05) in treated cells (Figure 6A) when compared to the control. The staphyloxanthin inhibition was also observed visually in myrtenol treated cells (Figure 6B).

### Effect of Myrtenol on the Susceptibly of MRSA to Human Blood and H_2_O_2_

The sensitivity of MRSA to healthy human blood and H_2_O_2_ was assessed between myrtenol (75, 150, and 300 μg ml^–1^) treated cells and the untreated cells by blood survival assay and H_2_O_2_ sensitivity assay. The results demonstrated that the myrtenol (300 μg ml^–1^) treated cells were highly susceptible to H_2_O_2_ (1.7 × 10^7^ cells) (Figure 6C) and healthy human blood (5 × 10^6^ cells) (Figure 6D) when compared to the control sample (3.5 × 10^7^ cells). Altogether, the resistance of MRSA against human blood and H_2_O_2_ was reduced upon treatment with myrtenol.

### Gene Expression Analysis

**FIGURE 7:**
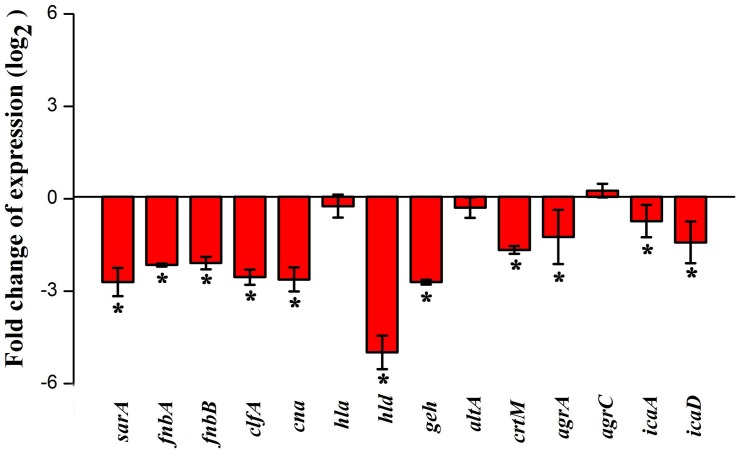
Effect of myrtenol treatment on expression of candidate genes involved in virulence factor production and biofilm formation of MRSA. Error bars indicate SD and asterisks indicate statistical significance (*p* ≤ 0.05).

Real-time PCR was performed to assess the effect of myrtenol at 300 μg ml^–1^ on genes involved in the virulence factors production and biofilm formation in MRSA. The results revealed down regulation in the expression of *sarA*, *agrA*, *icaA*, *icaD*, *fnbA*, *fnbB*, *clfA*, *cna*, *hla*, *hld*, *geh*, *altA*, and *crtM* and up regulation in the expression of *agrC* (Figure 7).

### Mature Biofilm Disruption

**FIGURE 8:**
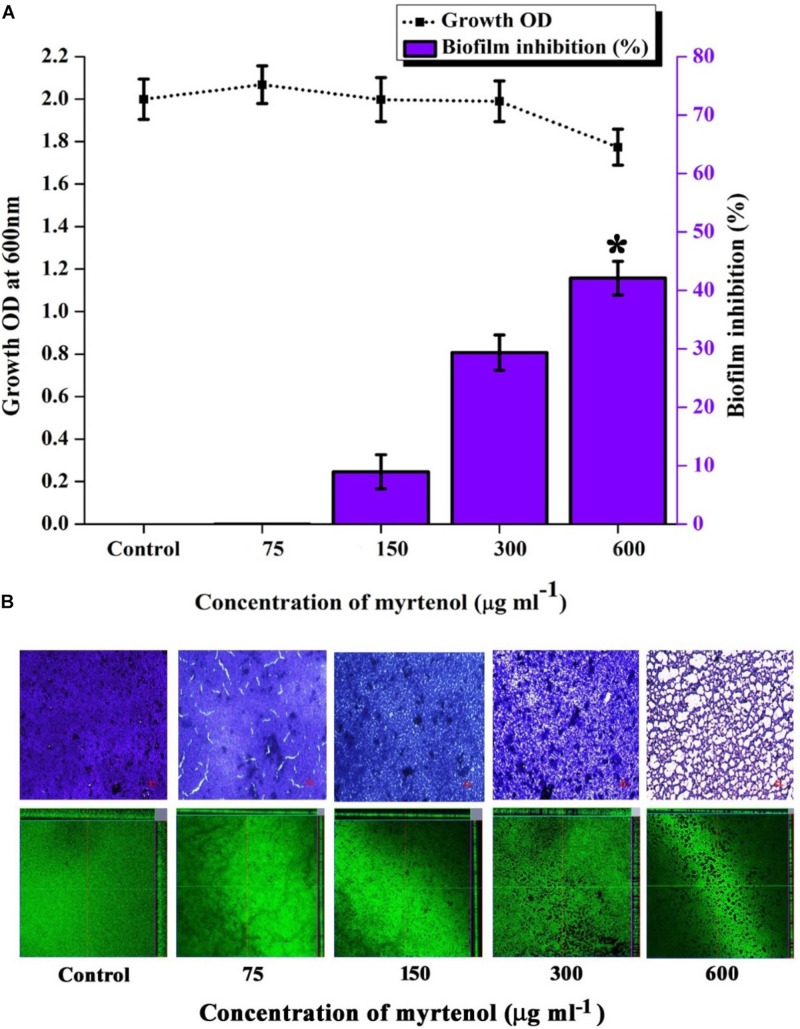
Effect of myrtenol treatment on preformed biofilm of MRSA. **(A)** Bar graph representing growth and biofilm disruption. **(B)** Microscopic analysis of mature biofilm disruption. Error bars indicate SD and asterisk indicates statistical significance (*p* ≤ 0.05).

Mature biofilm disruption assay was performed to assess the antibiofilm efficacy of myrtenol on the preformed MRSA biofilm. Growth of MRSA was found to be unaltered whereas no significant reduction in biofilm was observed at MBIC. Interestingly, at 600 μg ml^–1^ concentration, myrtenol disrupted 40% of the preformed biofilm of MRSA (Figure 8A). Spectrometric results were also confirmed by the light microscopic and CLSM analysis as well, in which the biofilm matrix was found to be reduced at 600 μg ml^–1^ concentration of myrtenol (Figure 8B).

### Analysis of Cytotoxic Effect of Myrtenol on PBMCs

The cytotoxic effect of myrtenol on PBMCs was assessed by trypan blue exclusion and Alamar blue assay. The fluorescent intensity of resazurin dye observed in the PBMCs with or without myrtenol (75, 150, 300, and 600 μg ml^–1^) was found to be unchanged (Supplementary Figure S5A). Trypan blue exclusion assay result revealed that myrtenol did not affect the viability of human PBMCs even at a concentration higher than MBIC for 24 h (Supplementary Figure S5B). Similar results were observed in the microscopic visualization of untreated and treated PBMCs where no significant reduction in viability or changes in morphology were observed when compared to the H_2_O_2_ treated positive control cells (Supplementary Figure S6). Hence, from the results it is apparent that myrtenol has no cytotoxic effect at the tested concentrations.

## Discussion

The present study for the first time revealed the antibiofilm and antivirulence efficacy of myrtenol against the clinically important pathogen MRSA by screening various phytochemicals. Myrtenol was found to be effective in reducing the biofilm forming ability of clinical isolates, namely MRSA 395, MRSA 410, and MRSA 44 in addition to MRSA ATCC 33591. Myrtenol exhibited a concentration dependent antibiofilm efficacy against all tested strains of MRSA with maximum reduction in biofilm at MBIC (300 μg ml^–1^) without affecting growth. However, at higher concentrations beyond 300 μg ml^–1^, growth of MRSA started to get affected, and complete growth inhibition was observed at 600 μg ml^–1^ and hence the same concentration was considered as MIC of myrtenol. Light and CLSM micrographs further substantiated the concentration dependent antibiofilm efficacy of myrtenol against all tested strains. As broadness of antibiofilm efficacy of myrtenol against clinical isolates was confirmed, MRSA ATCC strain alone was taken for further assays. Apart from surface biofilm, air-liquid interface biofilm formation provides ecological opportunity based benefits for aerobic bacteria to access both the aeration and nutrients in liquid media (Spiers et al., 2003; Robertson et al., 2013). Surface biofilm formation on polystyrene surface and air-liquid interface ring biofilm formation on glass surface was greatly diminished in the presence of myrtenol. An ideal antibiofilm agent is not expected to affect the growth and metabolic activity of the organism in order to exclude the development of resistance. Growth curve analysis of MRSA treated with 300 μg ml^–1^ of myrtenol exhibited the non-antibacterial nature of myrtenol. The cell viability was assessed by Alamar blue, a non-fluorescent blue dye that is reduced to the pink-color by the redox enzymes of metabolically active cells. Alamar blue assay confirmed that myrtenol does not affect the metabolic viability of cells. In addition, CFU also showed insignificant changes in the number of colonies between control and treated samples. *S. aureus* produces slime as an extracellular substance to facilitate the bacterial adhesion and biofilm formation. Slime production in *S. aureus* increases the ability of bacterial colonization on host tissue and enhances the persistence of bacteria and develops resistance to host immune responses (Ammendolia et al., 1999; Podbielska et al., 2010; Milanov et al., 2010). Several reports are available for the inhibition of slime synthesis by antibiofilm agents (Gowrishankar et al., 2012; Ansari et al., 2015; Rubini et al., 2018b). In our study we used CRA plate assay to qualitatively assess the impact of myrtenol on slime production. Decrement of black coloration around the colonies in myrtenol treated plates suggests the ability of myrtenol to reduce the slime production in MRSA.

Further, *S. aureus* produces a plethora of extracellular virulence factors and enzymes to invade and establish infections in the host system. Lipase is one such extracellular enzyme produced by *S. aureus* to interfere with the host granulocytes and bactericidal lipids. Thereby, lipase confers persistence and pathogenesis to *S. aureus*. In previous studies, lipase production has been demonstrated to enhance the biofilm formation in *S. aureus* (Rayner et al., 2012; Cadieux et al., 2014). As evident from lipase assay, myrtenol treatment substantially affected the production of lipase. Cytotoxins are also secreted virulence factors of *S. aureus* which play an important role in the establishment of infections. The α-hemolysin is one such kind of β-barrel pore-forming exotoxin which is highly potent in lysis of various host cells such as erythrocytes, macrophages, monocytes, endothelial cells and epithelial cells. It also has been reported as an important virulence factor in *S. aureus* to cause skin infection, septicemia, and pneumonia (Berube and Wardenburg, 2013; Chua et al., 2014; Tavares et al., 2014). Notably, hemolysin production by MRSA and clinical isolates was greatly inhibited by myrtenol treatment. Apart from secreted virulence factors, some structural proteins also contribute to the pathogenesis and adherence of *S. aureus*. Autolysin is a cell wall associated protein synthesized from the gene *altA* and involved in daughter cell separation, cell wall homeostasis, peptidoglycan layer turnover and autolysis (McCarthy et al., 2015). In a previous study, it was demonstrated that eDNA release in *S. aureus* has been mediated by the autolysis and also *altA* mutant *S. aureus* strain showed decreased eDNA release and biofilm formation (Houston et al., 2011). eDNA is reported to be abundant in *S. aureus* biofilm and involved in improving the structural stability of biofilm, horizontal gene transfer and development of antibiotic resistance (Kaplan et al., 2012). Therefore, autolysis and eDNA release in myrtenol treated samples were investigated and it was found that myrtenol significantly reduced the autolysis and release of eDNA. Being a major component of MRSA biofilm, eDNA has been reported to mediate intracellular adhesion which results in autoaggregation. Autoaggregation property protects *S. aureus* from antibiotics and also enhances the biofilm formation (Haaber et al., 2012). Hence, results of autolysis and eDNA quantification assays prompted to check the effect of myrtenol on autoaggregation. As expected, a concentration-dependent reduction in autoaggregation of MRSA was observed upon myrtenol treatment. In addition, myrtenol at the MBIC completely dispersed the MRSA when compared to autoaggregated cells of control sample.

An antioxidant production in *S. aureus* is essential for its growth and survival against reactive oxygen species (ROS) and also against the innate immune system of host organisms. Staphyloxanthin, a golden yellow colored carotenoid pigment, is an antioxidant and promotes *S. aureus* resistance to ROS (Clauditz et al., 2006). Inhibition of staphyloxanthin biosynthesis in *S. aureus* enhances its susceptibility to ROS and healthy human blood (Hall et al., 2017). Interestingly, in all the experiments performed, myrtenol treated cells appeared white in color when compared to golden yellow colored control cells. Thus, staphyloxanthin extracted from control and treated cells was quantified and it was confirmed that myrtenol treatment interrupts staphyloxanthin biosynthesis. In a previous report, rhodomyrtone, a staphyloxanthin inhibitor treated *S. aureus*, was shown to be more sensitive to H_2_O_2_ and human blood cells, which suggests that targeting staphyloxanthin inhibition is one of the alternative therapeutic strategies to control *S. aureus* infections (Leejae et al., 2013). Thus, the present study analyzed the susceptibility of MRSA to H_2_O_2_ and healthy human blood and found that myrtenol treated cells were more sensitive to H_2_O_2_ and healthy human blood when compared to control cells. These results further validated the staphyloxanthin inhibitory potential of myrtenol which enhanced the susceptibility of MRSA toward ROS and innate immune system.

In order to analyze the molecular mechanism of myrtenol, the gene expression profiles of control and myrtenol treated cells were studied using qPCR analysis. *sarA* is a well-known global master regulator of biofilm and virulence genes in *S. aureus*. Mutation in *sarA* is reported to alter the expression of 120 genes in which 76 genes are up regulated and 44 genes are down regulated (Dunman et al., 2001). Results of gene expression study revealed the down regulation of *sarA* upon myrtenol treatment. As the expression of MSCRAMMs involved in the initial stage of biofilm formation including FnbA, FnbB, Cna and ClfA and major virulence genes *hla* and *hld* is well known to be regulated by *sarA* (Cheung et al., 2004; Arya et al., 2015), the effect of myrtenol on these genes was examined and found to be down regulated. *sarA* mediated down regulation of adhesion genes by myrtenol could be the underlying reason for impairment of biofilm formation. Down regulation of *hla* and *hld* is in line with the result of the hemolysis assay performed. In addition, intracellular adhesion genes *icaA* and *icaD* positively regulated by *sarA* and involved in slime synthesis are found to be down regulated upon myrtenol treatment which was substantiated by decreased slime synthesis in CRA plate assay. Apart from *sarA*, the expressions of accessory gene regulatory loci *agrA* and *agrC* were also assessed. Though myrtenol treatment slightly induced the expression of *agrC*, down regulation of *agrA* was observed. As *agrA* and *argC* work together to regulate the virulence gene expression, down regulation of *agrA* ultimately affects the regulatory circuit. *sarA* is reported to control the expression of important virulence genes either dependent or independent of *agr* regulatory system (Chien et al., 1999). Specifically, SarA binds to the promoter region of *agr* regulatory system to co-ordinate the virulence gene expression (Reyes et al., 2011). Thus, reduced expression of *sarA* along with *agrA* in the presence of myrtenol, massively interfered the pathogenesis of MRSA. *sarA* based biofilm inhibition of the present study goes well with the previous studies wherein *sarA* inhibitors were reported to inhibit biofilm of *S. aureus* (Arya et al., 2015; Balamurugan et al., 2015, 2017). Specifically, *sarA* has been identified to be the target for drug discovery and development against *S. aureus* (Chien et al., 1999).

Glycerol ester hydrolase gene (*geh*) is involved in the synthesis of lipase enzyme (Cadieux et al., 2014) and this was found to be down regulated and also confirmed by phenotypic lipase assay. Expression of *atlA* was found to be slightly affected in the presence of myrtenol. However, this alteration greatly impaired the synthesis of eDNA and autoaggregation as observed in phenotypic assays. Down regulation of *crtM*, which is responsible for the synthesis of antioxidant pigment staphyloxanthin, was observed with the myrtenol treatment (Clauditz et al., 2006; Leejae et al., 2013). Hence, a reduction in the expression of *crtM* resulted in impairment of staphyloxanthin synthesis and further sensitized the MRSA cells to H_2_O_2_ and healthy human blood. On the whole, transcriptional analysis revealed the *sarA* mediated antibiofilm and antivirulence potential of myrtenol against MRSA.

Preformed biofilm is much more difficult to eradicate and well known to enhance the resistance of bacteria by blocking the diffusion of antimicrobial agents (Feldman et al., 2018). As evidenced from crystal violet quantification and microscopic analysis, myrtenol was found to eradicate the MRSA mature biofilm (40%) at 600 μg ml^–1^concentration of myrtenol. Biofilm eradicating efficacy is the added advantage of myrtenol to be a promising antibiofilm agent against MRSA. It is essential to analyze the cytotoxicity to ensure the pharmaceutical safety of drug as therapeutic candidate (McGaw et al., 2014). The cytotoxic experiments confirmed the non-toxic nature of myrtenol against PBMCs. As seen in trypan blue assay, myrtenol treated cells are intact in shape and excluded dye whereas cells treated with 1 mM H_2_O_2_ as positive control appeared blue in color. In Alamar blue assay, fluorescent intensity of myrtenol treated cells was comparable to that of control thereby proving the non-toxic nature of myrtenol. From the findings of the present study, it is clear that myrtenol is non-toxic, non-antibacterial, biofilm inhibitory and can effectively inhibit MRSA biofilm and hence can be used as therapeutic regimen against *S. aureus* infections.

## Conclusion

In summary, the present study demonstrated the antibiofilm potential of myrtenol without affecting the cell viability of MRSA and its clinical isolates. It also inhibited the production of major virulence factors of MRSA such as lipase, hemolysin and staphyloxanthin. In addition, it affected the slime synthesis, autoaggregation, autolysis, and eDNA production in MRSA. Furthermore, myrtenol is able to disrupt the mature biofilm to a considerable extent (40%) at 600 μg ml^–1^. The result of qPCR analysis revealed that myrtenol targets the *sarA* mediated gene expression, which is also well correlated with the *in vitro* assays. The antibiofilm and antivirulence potential of myrtenol along with its non-cytotoxic effect implies myrtenol as a harmless and potential therapeutic agent for the treatment of biofilm mediated MRSA infections.

## Data Availability

All datasets generated for this study are included in the manuscript and/or the Supplementary Files.

## Author Contributions

SP and AS designed the study. AS, TJ and AV performed the experiments. AS analyzed the data, prepared the figures and tables, and wrote the manuscript. SP revised the manuscript. All authors have read and approved the final version of the manuscript.

## Conflict of Interest Statement

The authors declare that the research was conducted in the absence of any commercial or financial relationships that could be construed as a potential conflict of interest.

## Supplementary Material

FIGURE S1 Effect of phytochemicals on growth and biofilm formation of MRSA. Error bars indicate SD and asterisks indicate statistical significance (*p* ≤ 0.05).

FIGURE S2 Effect of myrtenol on CFU of MRSA. Error bars indicate SD and asterisks indicate statistical significance (*p* ≤ 0.05).

FIGURE S3 Inhibitory effect of myrtenol on lipase synthesis and hemolysin production by MRSA. Error bars indicate SD and asterisks indicate statistical significance (*p* ≤ 0.05).

FIGURE S4 Effect of myrtenol on hemolysin production of MRSA clinical isolates. Error bars indicate SD and asterisks indicate statistical significance (*p* ≤ 0.05).

FIGURE S5 Cytotoxic effect of various concentrations of myrtenol on human PBMCs. **(A)** Alamar blue assay and **(B)** Trypan blue exclusion assay. Error bars indicate SD and asterisk indicates statistical significance (*p* ≤ 0.05).

FIGURE S6 Light microscopic images of PBMCs control, positive control and myrtenol treatment (Red color arrows indicate dead or dye included cells).

TABLE S1 List of primers used for qPCR analysis.

TABLE S2 Thermal cyclic conditions used for qPCR analysis.
