# Supplementary figures and images for: Myrtenol Attenuates MRSA Biofilm and Virulence by Suppressing sarA Expression Dynamism
Source: Front Microbiol. 2019 Sep 4;10:2027. doi: 10.3389/fmicb.2019.02027 (PMC6737500; doi:10.3389/fmicb.2019.02027)

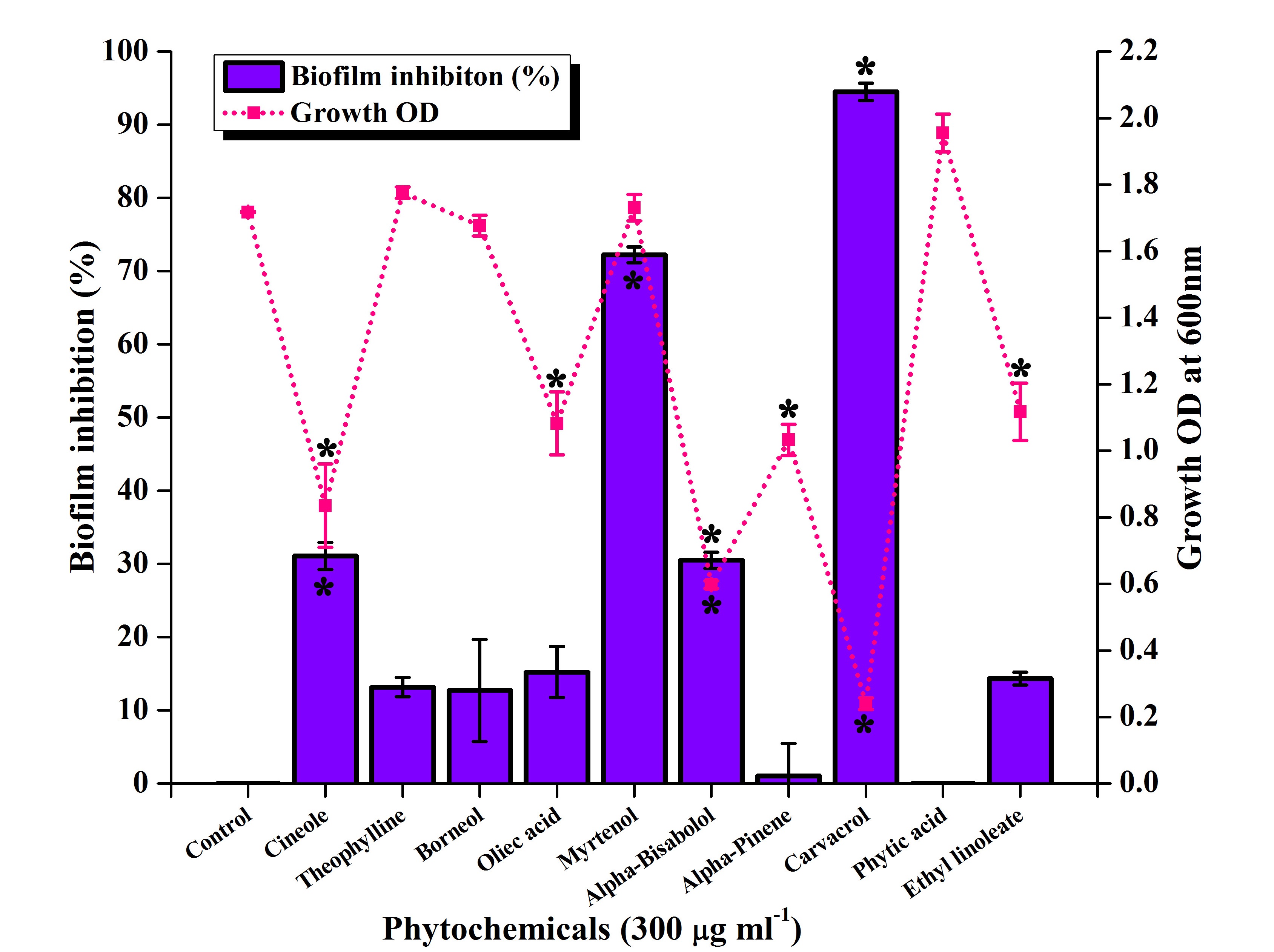

Supplement: FIGURE S1 — Effect of phytochemicals on growth and biofilm formation of MRSA. Error bars indicate SD and asterisks indicate statistical significance (p ≤ 0.05). [file Image_1.JPEG]

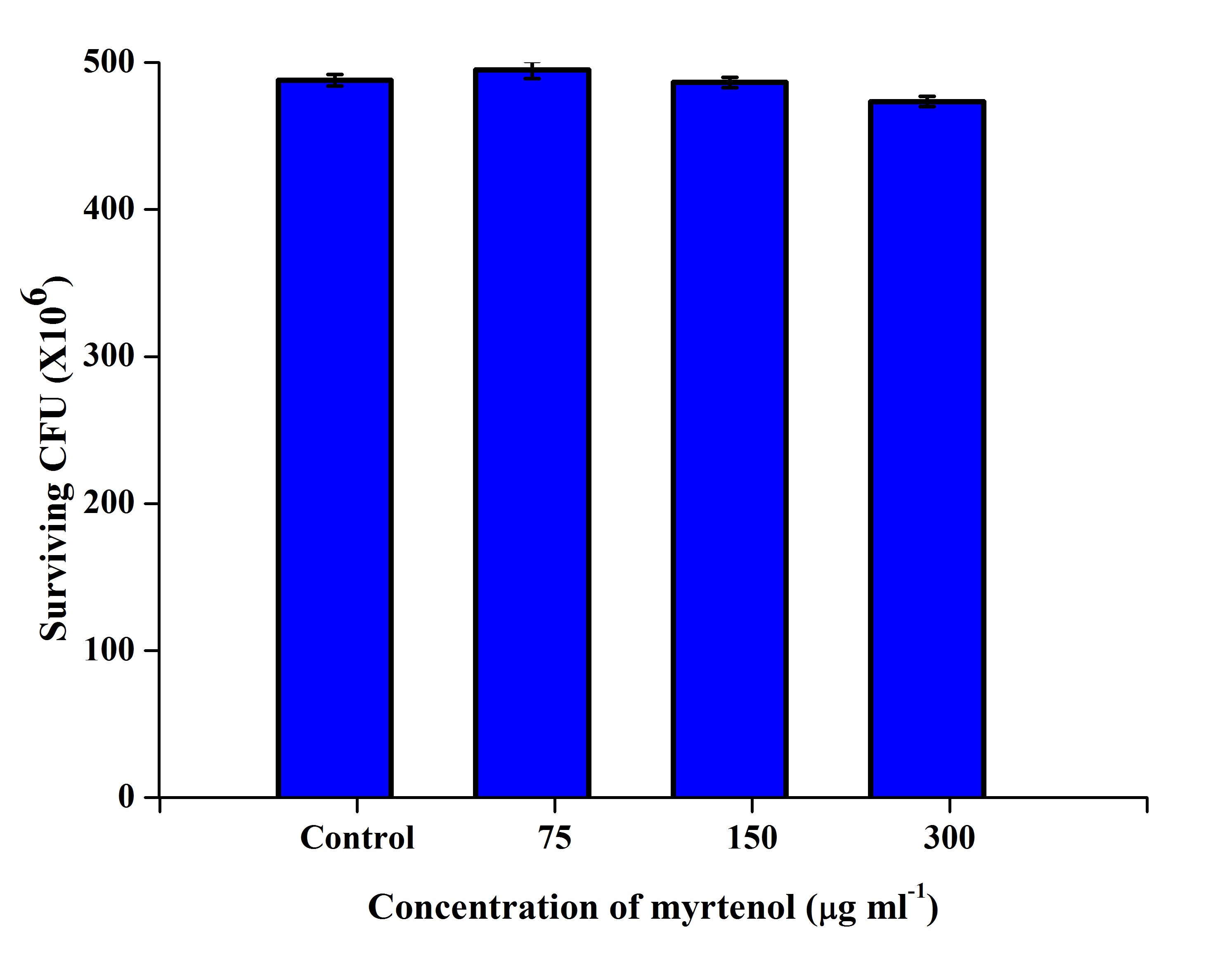

Supplement: FIGURE S2 — Effect of myrtenol on CFU of MRSA. Error bars indicate SD and asterisks indicate statistical significance (p ≤ 0.05). [file Image_2.JPEG]

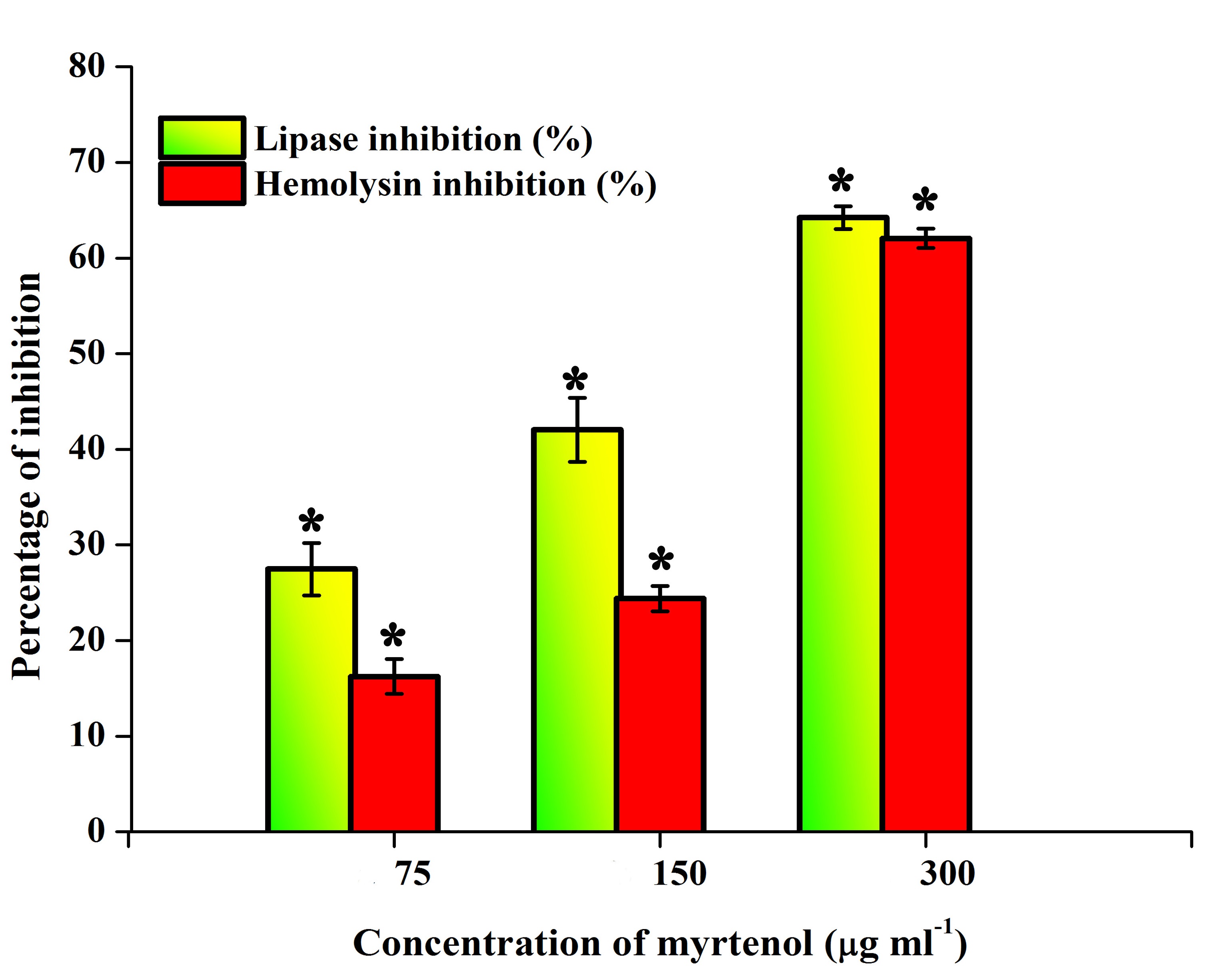

Supplement: FIGURE S3 — Inhibitory effect of myrtenol on lipase synthesis and hemolysin production by MRSA. Error bars indicate SD and asterisks indicate statistical significance (p ≤ 0.05). [file Image_3.JPEG]

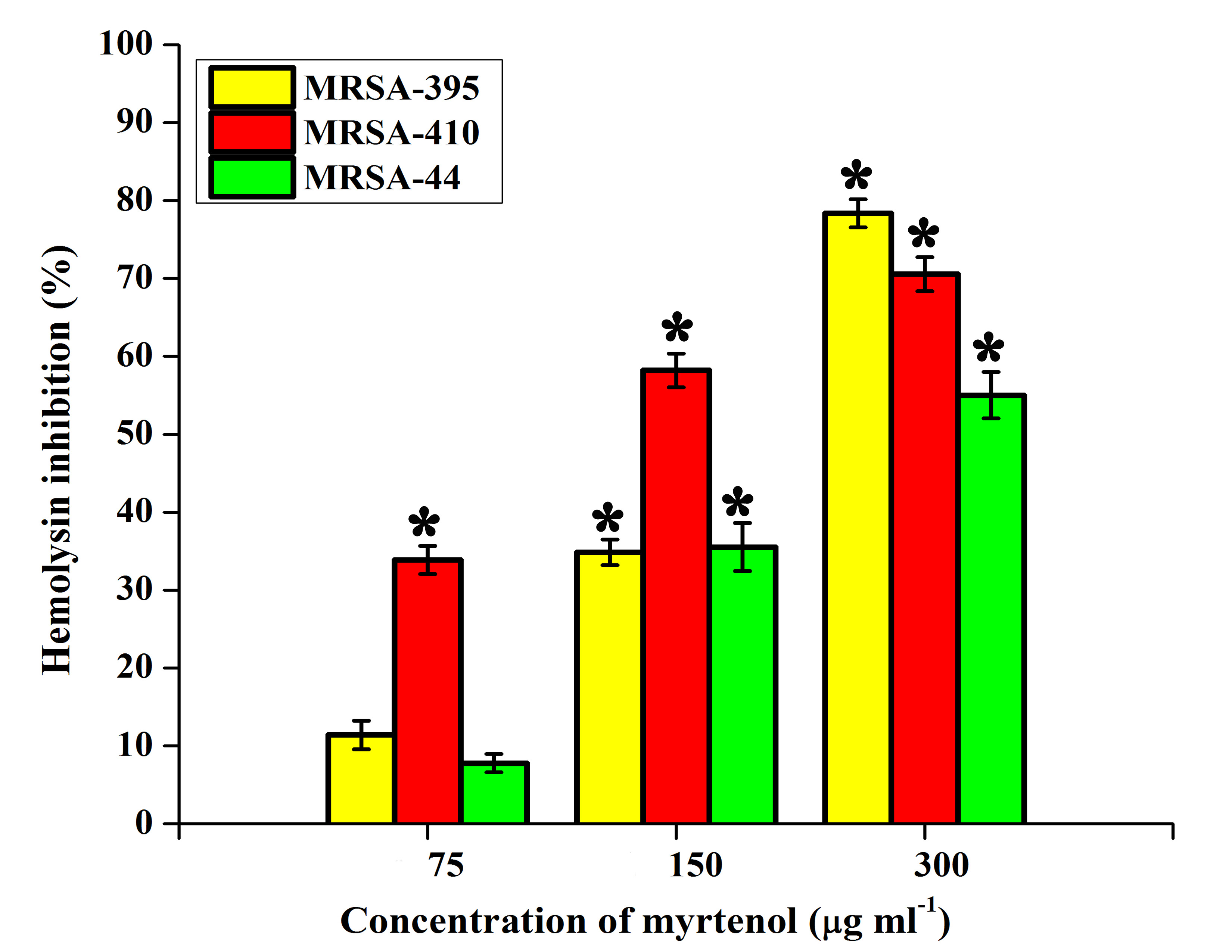

Supplement: FIGURE S4 — Effect of myrtenol on hemolysin production of MRSA clinical isolates. Error bars indicate SD and asterisks indicate statistical significance (p ≤ 0.05). [file Image_4.JPEG]

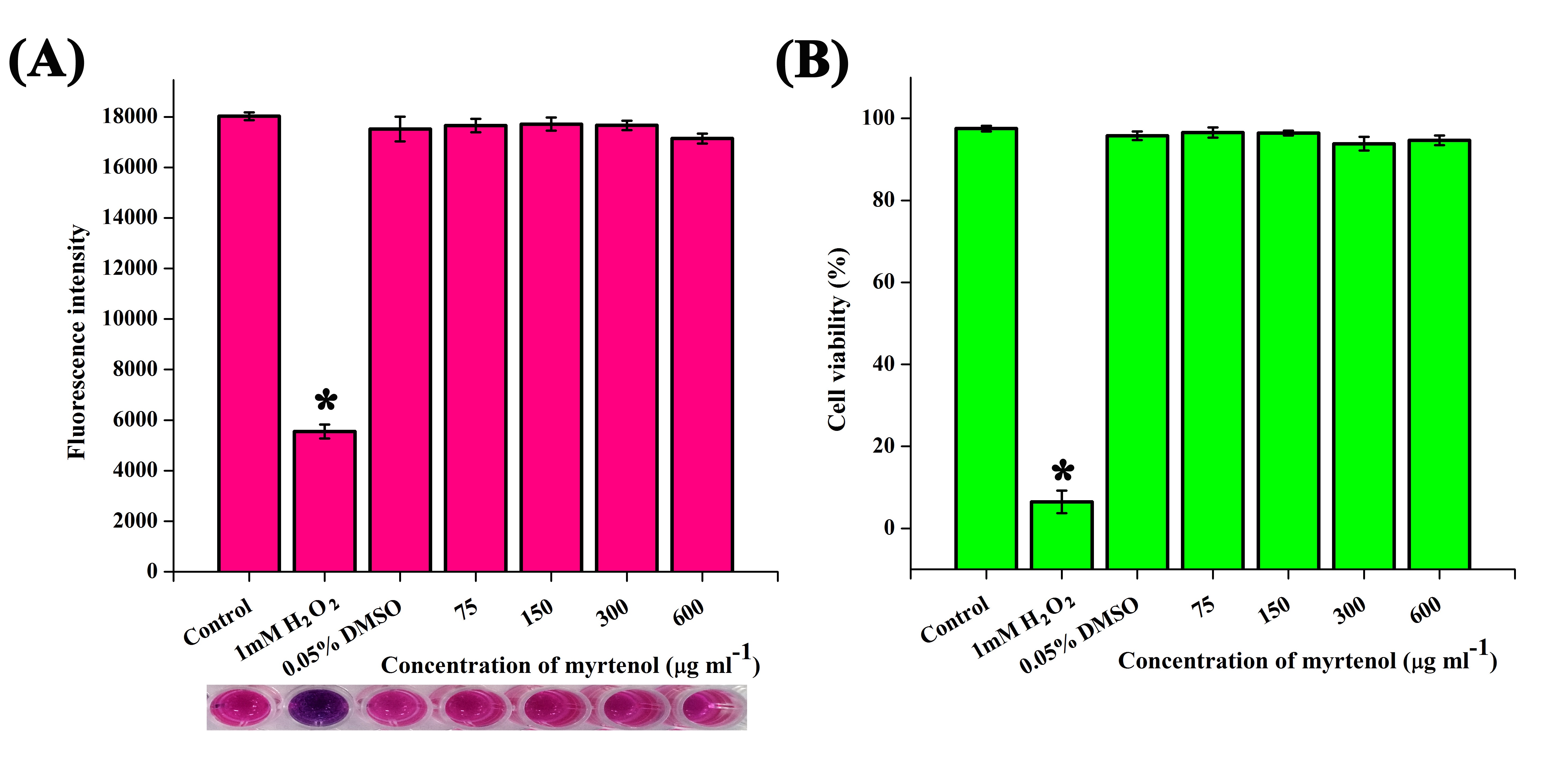

Supplement: FIGURE S5 — Cytotoxic effect of various concentrations of myrtenol on human PBMCs. (A) Alamar blue assay and (B) Trypan blue exclusion assay. Error bars indicate SD and asterisk indicates statistical significance (p ≤ 0.05). [file Image_5.JPEG]

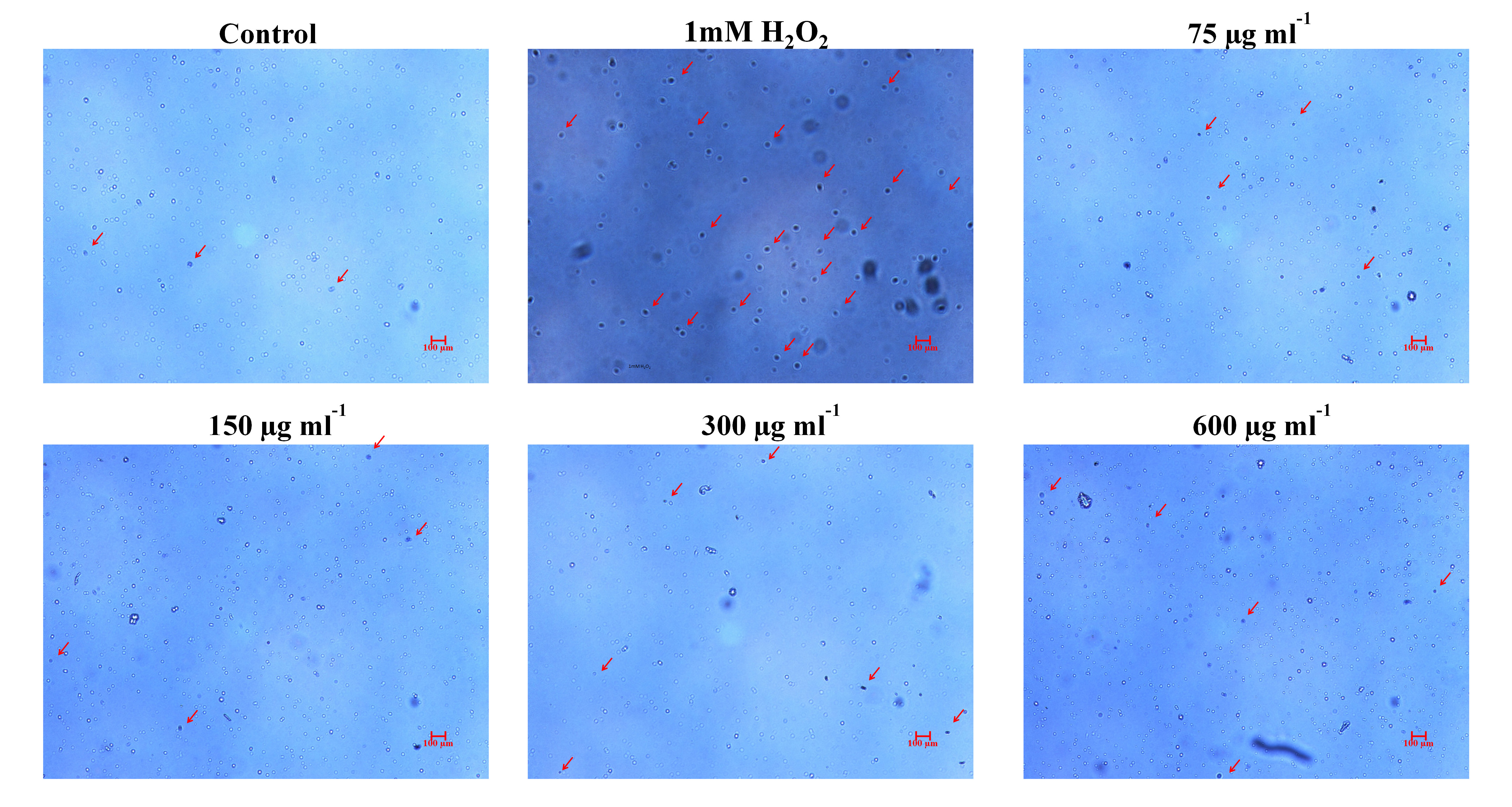

Supplement: FIGURE S6 — Light microscopic images of PBMCs control, positive control and myrtenol treatment (Red color arrows indicate dead or dye included cells). [file Image_6.JPEG]
