# Supplementary material for: Myrtenol Attenuates MRSA Biofilm and Virulence by Suppressing sarA Expression Dynamism
Source: Front Microbiol. 2019 Sep 4;10:2027. doi: 10.3389/fmicb.2019.02027 (PMC6737500; doi:10.3389/fmicb.2019.02027)
Supplement: TABLE S1 — List of primers used for qPCR analysis. [file Table_1.DOCX]

Supplementary Table 1. List of primers used for qPCR analysis.

| Genes | Forward primer | Reverse primer |
| --- | --- | --- |
| *gyrB* | 5'-GGTGCTGGGCAAATACAAGT-3' | 5'-TCCCACACTAAATGGTGCAA-3' |
| *sarA* | 5'-CAAACAACCACAAGTTGTTAAAGC-3' | 5'-TGTTTGCTTCAGTGATTCGTTT-3 |
| *fnbA* | 5'-ATCAGCAGATGTAGCGGAAG-3' | 5'-TTTAGTACCGCTCGTTGTCC-3' |
| *fnbB* | 5'-AAGAAGCACCGAAAACTGTG-3' | 5'-TCTCTGCAACTGCTGTAACG-3' |
| *clfA* | 5'-ATTGGCGTGGCTTCAGTGCT-3' | 5'-CGTTTCTTCCGTAGTTGCATTTG-3' |
| *cna* | 5'-AAAGCGTTGCCTAGTGGAGA -3' | 5'-AGTGCCTTCCCAAACCTTT-3' |
| *hla* | 5'-CAACTGATAAAAAAGTAGGCTGGAAAGTGAT-3' | 5'-CTGGTGAAAACCCTGAAGATAATAGAG-3' |
| *hld* | 5'-TAATTAAGGAAGGAGTGATTTCAATG-3' | 5'-TTTTTAGTGAATTTGTTCACTGTGTC-3' |
| *geh* | 5'-GTAGATTATGGCGCAGCACA-3' | 5'-CCATGCGCTTTATGATAGGC-3' |
| *altA* | 5'-TGTCGAAGTATTTGCCGACTTCGC-3’ | 5'-TGGAATCCTGCACATCCAGGAAC-3' |
| *crtM* | 5'-ATCCAGAACCACCCGTTTTT-3' | 5'-GCGATGAAGGTATTGGCATT-3 |
| *icaA* | 5'-ACACTTGCTGGCGCAGTCAA-3' | 5'-TCTGGAACCAACATCCAACA-3 |
| *icaB* | 5'-ATGGTCAAGCCCAGACAGAG-3' | 5'-AGTATTTTCAATGTTTAAAGCA-3' |
| *agrA* | 5′-TGATAATCCTTATGAGGTGCTT-3′ | 5′-CACTGTGACTCGTAACGAAAA-3′ |
| *agrC* | 5'-CATTCGCGTTGCATTTATTG-3' | 5'-CCTAAACCACGACCTTCACC-3' |
