# Supplementary material for: Myrtenol Attenuates MRSA Biofilm and Virulence by Suppressing sarA Expression Dynamism
Source: Front Microbiol. 2019 Sep 4;10:2027. doi: 10.3389/fmicb.2019.02027 (PMC6737500; doi:10.3389/fmicb.2019.02027)
Supplement: TABLE S2 — Thermal cyclic conditions used for qPCR analysis. [file Table_2.DOCX]

Supplementary Table 2. Thermal cyclic conditions used for qPCR analysis

| Initial denaturation | 95°C for 10 min | 40 cycles |
| --- | --- | --- |
| Denaturation | 95°C for 45 sec |  |
| Annealing | 58°C for 45 sec |  |
| Extension | 72°C for 45 sec |  |
